# The acceptability of smokeless tobacco products depends on nicotine levels

**DOI:** 10.1016/j.abrep.2019.100217

**Published:** 2019-08-27

**Authors:** R. Cruz-Cano, M. Rangel-Gomez, C. Van Wagoner, A. Kidanu, M.C. Brinkman, P.I. Clark

**Affiliations:** a University of Maryland, College Park; b Ohio State University

## Abstract

Understanding the role nicotine plays in initiating and sustaining addiction has been of interest for the scientific community and general population, with the idea that low levels of nicotine will reduce abuse liability associated with smokeless tobacco products. Previously, research has relied on subjective assessments to determine consumer acceptability, but these measures cannot provide a characterization of the physiological responses associated with nicotine use. Consumer acceptability arises from psychological and neurophysiological factors, thus establishing the need to use subjective and objective measurements in conjunction. This study provides a comprehensive characterization of the subjective and objective effects of smokeless tobacco product use with varying levels of nicotine. EEG data were recorded before and after the use of four different smokeless tobacco products and one control product over five separate visits, with participants arriving to each visit after 12 h of tobacco abstinence. These products have distinct consumer acceptability levels and patterns of use characteristics ranging from starter products to those used primarily by established users. Subjective results showed that smokeless tobacco products with higher levels of nicotine were more successful in reducing craving and more reinforcing than those with lower levels. These results were concordant with the activity present in the EEG recordings where products with high nicotine levels produced larger changes in the amplitude of the event-related signal than those with low levels. This study is fundamental in understanding the relationship between subjective and objective smokeless tobacco acceptability measurements, as mediated by the different levels of nicotine in each product.

## Highlights

• Smokeless tobacco acceptability depends on the level of nicotine.
• Nicotine levels relate to subjective reduction in tobacco craving.
• Event-related oscillations indicate that nicotine reduces effects of nicotine withdrawal.
• Nicotine abstinence alters brain's general state of preparedness.
• High levels, but not low levels, bring the brain to baseline activity levels.

## 1 Introduction

Nicotine is the main substance explaining the addiction to tobacco products (Markou, 2008), and to smokeless tobacco products in particular. Our research group has already demonstrated that the combined use of electrophysiological and subjective methods provides a more comprehensive evaluation of consumer acceptability than subjective data alone. While we have validated that electroencephalograms (EEG) can objectively quantify psychophysiological changes linked to product use, there is still a lack of objective evidence that quantifies the role nicotine content might have on consumer acceptability (Pritchard, 1991).

EEG data reliably indexes the changes that nicotine elicits in the brain (Conrin, 1980; Pritchard, 1991), but results have not been conclusive in the time domain (also known as Event Related Potentials – ERPs). Some studies have found that nicotine withdrawal reduces overall attentional resources in the brain (Evans et al., 2013) and that smokers show higher attentional allocation to smoking cues than to neutral ones (Oliver, Jentink, Drobes, & Evans, 2016) (as indexed by the P3 ERP component (Polich, 2007)). Other studies have not found such attentional modulation due to nicotine (Ascioglu, Dolu, Golgeli, Suer, & Ozesmi, 2004; Lindgren, Stenberg, & Rosen, 1998; Lv et al., 2016). The inconclusive results coming from ERP analyses have motivated researchers in the field to explore oscillatory patterns in nicotine-related neural responses (another analytical approach widely present in the field of psychophysiology) (Basar, Basar-Eroglu, Karakas, & Schurmann, 2001; Cui et al., 2013; Rangel-Gomez et al., 2019). Results using this approach have shown more consistent results than ERP analysis and, thus, is proposed in the present study.

Nicotine modulates the oscillatory activity in several frequency bands, but its effects are particularly salient in the alpha (8 to 13 Hz) frequency band. Activity in this band has been functionally linked to selective attention (in a task-dependent manner) (Klimesch, 2012) and arousal (in connection to the default network/resting state) (Mantini, Perrucci, Del Gratta, Romani, & Corbetta, 2007). In previous studies, enhanced allocation of attention has been linked to increased product acceptability (Buzzell et al., 2016), so we aim to provide support to the idea that this relationship is mediated by nicotine.

Nicotine modulates arousal and attentional resources, shifting the activity of the alpha band from a selective memory mode to a functional role in stimulus evaluation (from low to high alpha) (Lindgren et al., 1998). Nicotine-modulated changes in neural activation have been researched by manipulating the nicotine levels in combustible tobacco. High levels of nicotine lead to enhancements in arousal and altered allocation of attention, giving extra cognitive processing to tobacco related cues and elements (Domino & Matsuoka, 1994). Overall, the analysis of oscillatory activity (especially in low frequencies like theta and alpha) allows us to determine what level of nicotine will lead to negligible changes in general neural activity, which in principle can be related to lower potential for addiction.

The addiction potential of tobacco products depends largely on the amount and absorption speed of nicotine in the body (Hatsukami, Ebbert, Feuer, Stepanov, & Hecht, 2007). The nicotine in smokeless tobacco products is primarily present in two forms: monoprotonated and unprotonated. Because nicotine is a weak base, the fraction of any one form delivered to the user can be increased or decreased by using additives to manipulate the acidity or pH of the product (Tomar & Henningfield, 1997). The unprotonated, or “free-base” form is more easily absorbed in the mouth (United States. Advisory Committee to the Surgeon General., United States. Public Health Service, 1986), and users of smokeless tobacco products with higher levels of free-base nicotine exhibit greater dependence (Tomar, Giovino, & Eriksen, 1995). In addition to nicotine, the tobacco-specific nitrosamines (TSNAs) *N*′-nitrosonornicotine and 4-(methylnitrosoamino)-1-(3-pyridyl)-1-butanone are among the most harmful ingredients in smokeless tobacco products (Stepanov & Hatsukami, 2016). The Swedish National Food Agency Directive has regulated the contents of TSNAs and other toxicants in snus and chewing tobacco since 2016 (Rutqvist, Curvall, Hassler, Ringberger, & Wahlberg, 2011). The resulting reduced levels of TSNAs may explain the lack of association between snus use and oral cancer in Scandinavian countries (Hatsukami et al., 2015; Rutqvist et al., 2011). The concentrations of TSNAs and nicotine vary widely across conventional U.S. smokeless products, and are largely determined by conditions that can be controlled by the manufacturer during the growing and processing of tobacco (Hatsukami et al., 2015; Stepanov & Hatsukami, 2016). Therefore, establishing and implementing concentration limits for TSNAs and nicotine, or product standards, has great potential to reduce the public health toll of smokeless tobacco use in the US. (Hatsukami et al., 2015; Stepanov & Hatsukami, 2016).

As mentioned above, previous results from our group have linked ERPs and oscillatory activity (mainly in the alpha band) with attention and arousal, which relates to how much consumers accept certain products (Buzzell et al., 2016). Consumers' acceptability directly influences the likelihood that they will continue to use the product (Chassin, Presson, Sherman, & Margolis, 1988; Chassin, Presson, Sherman, McLaughlin, & Gioia, 1985), thereby contributing to the abuse liability and potential health risks from product use. Consumer acceptability is traditionally assessed using subjective measures (such as self-reported ratings) after product use (Hatsukami et al., 2011; Hatsukami, Zhang, O'Connor, & Severson, 2013). To our knowledge, no link has been made between product acceptability and nicotine level using a large spectrum of oscillatory (low alpha, high alpha and delta) activity in the electrophysiological signal in response to various nicotine levels.

In the present study, we related the subjective measurements of product acceptability with the activity in event-related oscillations at different frequency bands, in relation with the effects of different levels of nicotine in the neural activity (as indicated by the electroencephalograms recorded during resting state). Previous studies have used cognitive tasks as a conduit to analyze changes in neural activity due to nicotine. The resting state has become a pivotal tool in understanding general neural resources/states given that it provides a characterization of the baseline brain resources. When external stimulation (such as nicotine) is administered, the effects are not confounded by the interaction between the task specifics and the type of stimulation applied (i.e., some tasks are more susceptible to changes due to nicotine).

On five separate occasions, participants were asked to come into the lab and received smokeless tobacco products of different nicotine levels and tobacco cuts (i.e. long cut that consist of long strands, fine cut that consists of moist snus, and a moist snus contained in a sachet). We hypothesized that products with higher levels of nicotine would be accepted to a greater extent and that this acceptability would be related to higher scores in the subjective measurements and larger enhancements in the oscillatory activity in the brain regardless of other characteristics of the products.

## 2 Methods

### 2.1 Participants

One female and twenty-seven male smokeless tobacco users (21 Caucasian, 4 mixed races, 2 African American and one Asian American) ranging in age from 18 to 62 years (Median Age = 21.2 years, Standard Deviation = 4.3 years) were recruited from the community around the University of Maryland, College Park and paid $400 dollars for the completion of five laboratory visits. All participants reported the use of smokeless tobacco for at least one year prior to study participation, no psychiatric or neurological conditions nor the use of medication for such conditions, and no reported use of psychoactive drugs (alcohol abstinence was asked 12 h before each visit). All participants provided written informed consent, and all procedures were approved by the Institutional Review Board of the University of Maryland, College Park, and performed in accordance with the Declaration of Helsinki.

### 2.2 Procedure

Self-reported tobacco abstinence of at least 12 h was required before each of the five separate laboratory visits. During each visit, smokeless tobacco users used one of four smokeless tobacco products (Copenhagen Long Cut Regular, General Swedish snus Classic Blend, Hawken Rough Wintergreen, Red Man Long Cut Wintergreen) or a Nicorette Nicotine Lozenge as a control, while subjective and objective measurements were taken before and after the administration of each of these products. Each laboratory visit occurred at least 24 h apart and around the same time per day to ensure the required tobacco abstinence period was met. Product administration was blinded to participants, and product order was counterbalanced across sessions and individuals. Participants were instructed to use the products for 30 min at which point any unused product was discarded. The amount of tobacco consumed (estimate of exposure) was not collected.

At the beginning of each visit, participants filled out a series of subjective questionnaires while lab assistants prepared the EEG cap on the participant's head (approximately 45 min). Once the EEG setup was complete, participants performed a resting state task immediately before and immediately after product administration (while EEG data was recorded). A nature documentary was shown during product consumption, but EEG data was not recorded due to numerous artifacts coming from the chewing of smokeless tobacco.

### 2.3 Smokeless tobacco products

All products were orally administered to participants. The smokeless tobacco products prepared as long cut (or loose leaf) tobacco were measured for mass (2 g) and given to participants in a plastic tin. The smokeless tobacco products prepared in sachets/pouches (General Classic Blend only) were given directly to participants with no modifications. The products administered to participants were the following (each on a separate visit): Red Man Long Cut Wintergreen (Swedish Match) unprotonated nicotine = 3.53 mg, total = 13.5 mg; Copenhagen Long Cut Regular (Altria) unprotonated nicotine = 3.78 mg, total = 11.2 mg; General Swedish snus Classic Blend (Swedish Match) unprotonated nicotine =4.29 mg, total = 7.19 mg; Hawken Rough Wintergreen (American Snuff Company/Reynolds American) unprotonated nicotine = 0.01 mg, total = 3.99 mg; and Nicorette nicotine lozenge “original” (Glaxo Smith Kline) unprotonated nicotine = 1.41 mg, total = 1.43 mg. Total nicotine was determined using gas chromatography with flame ionization detection (MacGregor et al., 2014), and the fraction that was in the free-base form was estimated from the measured total nicotine and pH of the product according to the Federal Register method (*Federal Register*, 1999; *Federal Register*, 2009). In addition to variable nicotine levels, the products chosen for this study presented some unique consumer acceptability characteristics. The two Swedish Match products (General Classic and Red Man) were chosen because General Classic is a Swedish snus and is thus a low TSNA product, and Red Man is not. Hawken is referred to as a “starter product” for smokeless tobacco because it has relatively low levels of total and free-base nicotine to minimize nausea, vomiting and dizziness in new smokeless tobacco users (Alpert, Koh, & Connolly, 2008). Copenhagen represents the opposite end of that spectrum, with high total and free-base nicotine content, and it is used primarily by established users (Alpert et al., 2008; Djordjevic, Hoffmann, Glynn, & Connolly, 1995). It contains almost 3 times the amount of total nicotine as Hawken, and 1/3 of that nicotine is in the free-base form, which is more rapidly bioavailable and produces a more intense “kick” or “rush” (Ashley, Pankow, Tavakoli, & Watson, 2009; Tomar & Henningfield, 1997).

### 2.4 Subjective scales

The demographic and tobacco use history questionnaire and a modified version of the Fägerstrom Test for Nicotine Dependence (mFTND) were completed on the first visit only. The remaining surveys were completed at different moments during all five laboratory visits. Participants completed a modified version of the Questionnaire on Smoking Urges (mQSU-brief) and of the Minnesota Nicotine Withdraw Scale (mMNWS) before and after product administration and a modified version of the Cigarette Evaluation Scale (mCES), also referred to as the “Cigarette Evaluation Questionnaire,” following product use, and the Duke Sensory Questionnaire (mDSQ) also following product usage. If required, questionnaires were modified for smokeless tobacco products (e.g., “smoke cigarettes” changed to “use smokeless tobacco”).

### 2.5 Resting state measurement

Participants were instructed to use smokeless tobacco products for 30 min; before and after this period, 12 ‘eyes-open' (resting state) recordings were done (6 before and 6 after). The first recording served as a baseline and was completed 90 s before product administration. During the resting state periods, participants were instructed to fixate on the middle of a completely blank screen while limiting movement and eye blinks.

### 2.6 Electrophysiological recording

EEG data were recorded using 16 Ag/AgCl scalp electrodes, placed following the extended 10/20 international system (FP1, FP2, F3, Fz, F4, T3, C3, Cz, C4, T4, P3, Pz, P4, O1, Oz, O2), which were embedded in an elastic cap. Electro-oculograms were obtained from electrodes placed on the left supraorbital and suborbital sites and the left and right outer canthal sites. The sampling rate for data collection was set at 500 Hz with a Neuroscan NuAmps amplifier and SCAN 4.01 software (Compumedics, North Carolina). The online reference was set to the left mastoid electrode and the offline reference was set to the average of the left and right mastoid electrodes. Impedances were kept below 10 kΩ in all electrodes and an online 70-Hz low-pass filter was used.

### 2.7 Analysis of subjective scales

The following factors were calculated for each of the subjective scales:

- mCES: “Total Reinforcement”, “Psychological Reward,” “Satisfaction”, “Enjoy Sensations”, “Craving Reduction” and “Aversion” factors were calculated after product usage.
- mMNWS: “Negative Affect”, “Craving”, and “Increased Appetite” and “Total Wthdrawal” factors were calculated before and after product usage; difference scores were computed.
- mQSU-brief: “Total Urge”, “Intention and Desire to use Smokeless Tobacco”, and “Relief of Negative Affect and Urgent Desire to use Smokeless Tobacco” factors were calculated before and after product usage; difference scores were computed.
- mDSQ: there were no factors calculated for this scale; instead, each of the items in the questionnaire (“Liking”, “Satisfaction”, “Perceived amount of Nicotine” and “Perceived Strength”) was captured, along with “Total Sensory Perception” following product usage.

Statistical analysis was performed using a separate repeated-measures analysis of variance (ANOVA) for each factor of the mDSQ, mMNWS, mCES and mQSU-brief. When needed *p*- values for all ANOVA models were Bonferroni-corrected.

### 2.8 Analysis of electrophysiological data

#### 2.8.1 Preprocessing

Raw EEG data were re-referenced to the average of the left and right mastoid electrode signals and linearly de-trended. A Butterworth low-pass filter was set to 30 Hz to remove high-frequency artifacts without altering frequencies of interest that were below this threshold. Each of the resting-state EEG datasets was divided into time-windows (epochs) of 1 s, leading to 90 epochs per block.

Artifact rejection was done in two steps; first, large artifacts were rejected using a ± 1000 μV threshold and second, large linear drifts within epochs were rejected using the pop_rejtrend function of the EEGLAB toolbox (Delorme & Makeig, 2004), with a maximum slope threshold of 75 μV and an R2 limit of 0.8. EMG activity was detected and rejected using the EEGLAB function pop_rejspec, using a 50 dB threshold within the 20–40 Hz band. Ocular artifacts were detected using independent component analysis (ICA) on a sample dataset high-pass filtered to 1 Hz. ICA component weights were applied to the remaining datasets and components corresponding to eye artifacts were rejected. Approximately 5% of the trials were removed due to artifacts.

### 2.9 EEG analysis

Power spectral density (PSD) was extracted from resting state EEG epochs using a 512-point hamming-windowed Fourier transform (frequency bin width = 0.98 Hz). Raw PSD (μV2/Hz) was logarithmically transformed to decibel (dB) PSD (10 × log10 [μV2/Hz]). Global alpha power was defined as PSD between bin centers 7.81 Hz and 12.70 Hz at electrodes F3, F4, C3, C4, P3, and P4. For each resting state period, global alpha power was averaged across epochs. To investigate how each product influenced global alpha power over time, alpha difference scores were calculated for each time point/product. Specifically, global alpha power before product usage was subtracted from global alpha power for each time point after usage. Changes in global alpha power, as a function of product type and time point, were analyzed using a repeated measures ANOVA model to test mean differences between the product types and time points.

### 2.10 Analyses of relationships between electrophysiology and subjective scales

Correlations between the electrophysiological measures and the subjective scales were tested. Separate Pearson product-moment correlations were calculated for the low alpha (*8 to 10 Hz*), high alpha (11–13 Hz, and delta difference scores, with the mCES, mMNWS, and mQSU-brief factors and the mDSQ items. For visualization and interpretation purposes, the sign of the negative values in the electrophysiological measures were reversed (i.e., an increased high alpha suppression would now register as a positive value).

## 3 Results

### 3.1 Demographic analysis

On average, participants reported using 2.45 tins (*n* = 21) or 16.43 pouches (*n* = 7) of smokeless tobacco per week for a mean of 2.48 years (*SD* = 19.06) on average. Participants scored a mean of 2.04 (*SD* = 1.64) on the Fägerstrom Test for Nicotine Dependence, indicating a low level of dependence. Of the 28 participants in this study, six reported currently smoking cigarettes, for which the mean number of cigarettes per day was 2.6 (range = 1–5 cpd). Twenty-two participants were former smokers.

### 3.2 EEG results

**Fig. 1:**
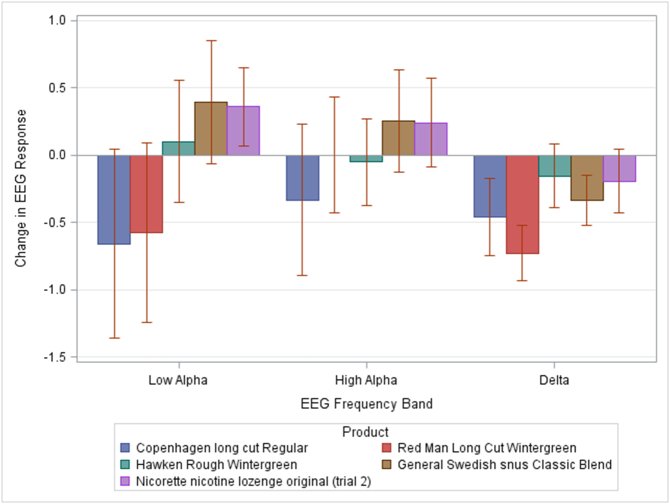
Low alpha, high alpha, and delta power difference scores as a function of product type. Difference scores reflect band power amplitude following product usage (collapsed across time point), minus band power prior to product usage. Error bars reflect the standard error of the mean.

Data belonging to all 28 participants were included for EEG analysis. Repeated Measures ANOVA was conducted to compare mean change in EEG responses (post-product response minus pre-product response) in the delta, low alpha, and high alpha bands, due to the effect of smokeless tobacco consumption (see Fig. 1). This analysis revealed a significant main effect of product type in low alpha (*F*(4,105) = 16.79, *p* < .0001, η^2^ = 0.0955), high alpha (*F*(4,105) = 5.28, *p* = .0006, η^2^ = 0.0327), and delta (*F*(4,105) = 13.69, *p* < .0001, η^2^ = 0.0782).

In *low alpha,* products with the highest nicotine content produced the largest pre-post change in power. Only the two products with the highest nicotine levels (Copenhagen Long Cut Regular and Red Man Long Cut Wintergreen) showed a post-product decrease in low alpha power, while all other products showed an increase in power. In *high alpha,* the effects of variable nicotine levels were not as localized as low alpha products with the three largest levels (Copenhagen Long Cut Regular, Red Man Long Cut Wintergreen and Hawken Rough Wintergreen) showing a similar pattern of higher pre- than post-product power. Interestingly, there was a clear non-gradual cutoff, with nicotine levels lower than 4 mg showing the opposite pattern (higher post- than pre-product power).

Regarding activity in the delta band, any level of nicotine produced the same pattern of change in the power in this frequency band (larger pre- than post-product). However, the difference is still the largest for products with the highest nicotine levels.

Incorporating variables related to Cigarette use, Years of smokeless products used, Type of smokeless product normally used, Frequency of use and Number of products used per day and/or all of the variables at the same time in the regression model led to results which are pretty much undistinguishable to those seen in Fig. 1 (Appendix 1).

### 3.3 Subjective results

**Fig. 2:**
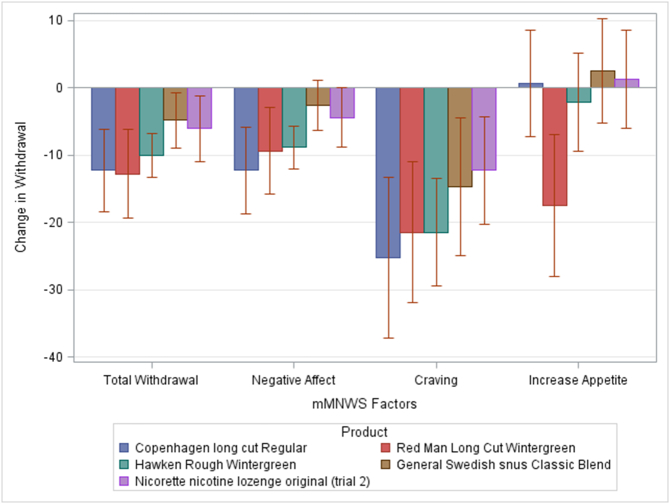
Change in factor scores for the modified version of the Minnesota Nicotine Withdrawal Scale (mMNWS) by product type. mMNWS factor scores reflect the difference between ratings taken after product usage minus ratings taken before product usage. Error bars reflect the standard error of the mean.

**Fig. 3:**
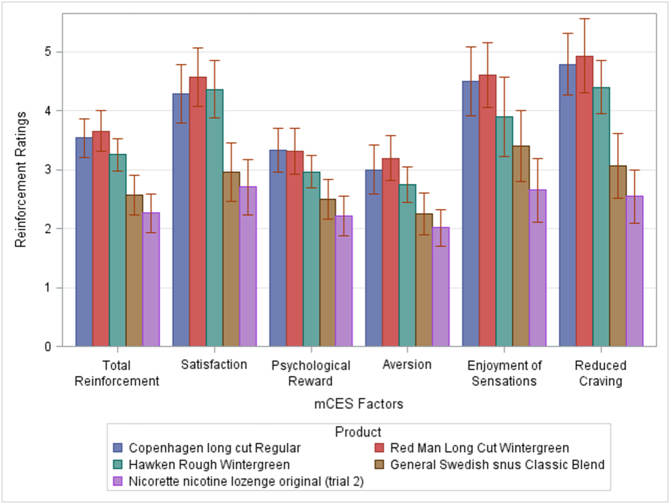
Mean ratings of total reinforcement and factor scores for the modified version of the Cigarette Evaluation Scale (mCES) by product type. mCES scores reflect ratings taken after product usage. Error bars reflect the standard error of the mean.

Repeated Measures ANOVA Analyses were conducted to compare mean change in subjective responses (post-product response minus pre-product response) to the five products (see Fig. 2, Fig. 3). Findings revealed several main effects of product type (Table 1).

**Table 1:** Repeated Measures ANOVA results for the subjective measures.

Subjective factor	Questionnaire	F test	*P* value	Partial Eta Squared
Total Urge to use smokeless tobacco	mQSU-brief	3.77	0.0065	0.11929
Total Withdrawal	mMNWS	4.18	0.0035	0.13112
Negative Affect from Withdrawal	mMNWS	3.94	0.0050	0.12339
Craving from Withdrawal	mMNWS	2.53	0.0450	0.08040
Increased Appetite from Withdrawal	mMNWS	5.26	0.0007	0.16360
Total Reinforcement	mCES	27.98	<0.0001	0.50191
Satisfaction	mCES	19.35	<0.0001	0.41054
Psychological Reward	mCES	15.56	<0.0001	0.35798
Aversion	mCES	13.59	<0.0001	0.33124
Enjoyment of Sensations	mCES	12.81	<0.0001	0.31563
Reduced Craving	mCES	25.49	<0.0001	0.47750
Total Sensory Perception	mDSQ	47.69	<0.0001	0.63110
Liking	mDSQ	15.70	<0.0001	0.35950
Satisfaction	mDSQ	25.87	<0.0001	0.48081
Perceived amount of Nicotine	mDSQ	40.26	<0.0001	0.59039
Perceived Strength	mDSQ	42.84	<0.0001	0.60646

Pairwise analyses revealed that the most statistically significant results are that Red Man Wintergreen moist snuff, Hawken Rough and Copenhagen moist snuff were rated as more reinforcing, and provide more satisfaction, reduce craving and psychological reward (mCES) than both the General Swedish snus, and the Nicorette lozenge (all adjusted *p*-value < .0001).

The same can be said for the total score of the mDSQ survey and its 4th item, “How strong do you think the tobacco was?” (all adjusted *p*-value < .0001). Red Man wintergreen moist snuff and Copenhagen moist snuff also score significantly higher than Hawken Rough for the 3rd item, “How high in nicotine do you think the tobacco that you just used is?” and 4th item of the mDSQ survey. Although there were statistically significant pairwise comparisons for mMNWS and mQSU-brief surveys (p-value < .05), their corresponding *p*-values were larger than 0.0001.

As it was the case for the EEG measures including variables related to Cigarette use, Years of smokeless products used, Type of smokeless product normally used, Frequency of use and Number of products used per day and/or all of the variables at the same time in the regression model led to results which are nearly identical to those seen in Fig. 2, Fig. 3 (Appendix 1).

### 3.4 Relationships between electrophysiological and subjective responses

Separate Pearson product-moment correlations were conducted to test the relationships between the electrophysiological and subjective responses to the five study products (see Table 1). Pearson's R values were calculated for the mean change (post – pre-product) of low alpha, high alpha, and delta power, in relation with the mean change in the following subjective responses: Total Withdrawal (mMNWS factor), Total Urge (mQSU-brief factor), Intention and Desire to use Smokeless Tobacco (mQSU factor), Relief of Negative Affect and Urgent Desire to use Smokeless Tobacco (mQSU-brief factor), Reinforcement (mCES factor), Satisfaction (mCES factor), Psychological Reward (mCES factor), and Aversion (mCES factor). Only ratings of the Reinforcement (mCES) scale presented significant correlations with the electrophysiological measurements (see Table 2).

**Table 2:** Pearson *R* correlations between electrophysiological and subjective responses.

		mCES factors	
	Total reinforcement	Satisfaction	Psychological reward
Low alpha	−0.189⁎	−0.129	0.190⁎
High alpha	−0.226⁎⁎	−0.199⁎	−0.233⁎⁎
Delta	−0.148	−0.069	−0.138

mCES = version of the Cigarette Evaluation Scale modified for Smokeless Tobacco use. To improve the interpretability of the correlations, the signs of Low Alpha, High Alpha, and Delta band difference scores were reversed.

⁎ *p* < .05.

⁎⁎ *p* < .01.

## 4 Discussion

The present study aimed to understand cognitive changes in different levels of nicotine in smokeless tobacco products and how those neural effects correlate with subjective measures of product acceptability. An important element of our study is that participants arrived at each of the five sessions after at least 12 h of nicotine abstinence providing a reference frame for the understanding our results. We hypothesized that high levels of nicotine would reduce changes in neural activation due to nicotine abstinence and, at the same time, reduce adverse effects of nicotine deprivation thus enhancing product acceptability.

Our results support these hypotheses, with pronounced changes in the electrophysiological responses of the brain during a resting state task for products with the highest levels of nicotine and not for products with low levels of nicotine. In the delta band, before product use, the oscillatory activity was enhanced in response to nicotine abstinence; in accordance with the idea that delta activity marks the connection between the default network and autonomic functions (Knyazev, 2012; Meerwijk, Ford, & Weiss, 2015), which modulates the expectation of reward (Wu et al., 2018), especially coming from the use of substances of abuse, such as the nicotine present in smokeless tobacco products. Products with high levels of nicotine managed to reduce such enhanced neural activation due to nicotine abstinence with the highest levels of nicotine producing the biggest changes in the activity in the delta band. This is concordant with subjective reports since the products containing the highest levels of nicotine also produced the greatest changes in withdrawal (as measured by the modified Minnesota Nicotine Withdrawal Scale) - (importantly associated with craving) and in reinforcement (as measured by the modified Cigarette Evaluation Questionnaire) (Cappelleri et al., 2007). Interestingly, acceptability or electrophysiological activity did not depend on patterns of use. Variations in these factors were related solely to the level of nicotine and not to other constituents in the products that drive more or less experienced users to prefer any given brand/type.

Regarding oscillatory activity, as the number of oscillations increases the activity correlates more specialized functions and recruits higher cognitive processes (Basar et al., 2001). In our study, the alpha band, which is related to arousal mediated by the connection between the default network and autonomic functions (Knyazev, 2012; Meerwijk et al., 2015) and to the preparation for selective attentional engagement in a task-related manner (Foster, Sutterer, Serences, Vogel, & Awh, 2017), showed such transition from less to more specialized activity. Reduced low alpha activity was seen in post-product administration of smokeless tobacco products, with the highest nicotine levels indicating a reduction in abstinence-related alpha enhancement related to reduced arousal. Products containing lower nicotine levels did not manage to reduce such enhancement. In high alpha activity, results did not follow the trend present in lower oscillatory activity (low alpha and delta), possibly suggesting that activity in higher frequencies is less modulated by nicotine. According to the theory that lower frequencies are related to more basic and topographically widespread brain functions (Cui et al., 2013), nicotine may influence arousal levels and non-voluntary reward expectation. On the contrary, more complex and region-localized functions are not affected by nicotine, such as attentional evaluation of the products used. The difference found for the Copenhagen snuff may be related to an evaluation of other components of these smokeless tobacco products and not necessarily related to nicotine.

These results are important because it shows that electrophysiological measurements adequately represent changes in the brain due to different levels of nicotine in smokeless tobacco products. Second, where subjective measurements are more general, time-frequency decomposition can identify different brain processes involved in product acceptability. This is confirmed by two sets of results. First, electrophysiological measurements show different trends in different frequency bands in varying levels of nicotine, whereas subjective measurements provide a global value. Therefore, electrophysiological measurements show that each frequency band is related to a specific cognitive process, and our results show that nicotine has an effect in the overall state of the brain at the arousal/preparatory level, which tends to dissipate as the cognitive processes required become more complex. Importantly, our second set of results (correlational analysis) show that the changes in neural activity captured by the widely used subjective questionnaires are evaluative in nature and that smaller changes in the neuro-activation associated, for example, with low levels of nicotine, may not be detected by such instruments.

In conclusion, our results show, for the first time, that acceptability to smokeless tobacco products is connected to activity in a diverse range of neural oscillatory activities and that this relationship is mediated by nicotine levels in such products. It also shows that this process is modulated by different functional specializations in the brain. Our participants arrived at each session after prolonged nicotine abstinence, so our study identified the changes from a state of withdrawal transitioning to satiety. The effects that the widely used subjective measurements detect can be objectively explained by means of electrophysiology and time-frequency analysis. These results are of central importance for the study of the effects of different levels of nicotine in tobacco products, and how to find a nicotine level that has negligible neural activation and therefore low addictive potential.

### 4.1 Limitations

Although our results are a pioneer in the detection of the effects of different levels of nicotine in smokeless tobacco products, we have identified two main limitations. First, our results are valid for smokeless tobacco, but this may not be true for other tobacco products that deliver nicotine in different ways. Second, although unlikely, our results may be confounded by differences in administered products because, even though all the products were smokeless tobacco, they varied in features such as flavor and cut size.

Results from previous studies in our group have indicated that nicotine is the main driver of differences in the oscillatory effects observed after tobacco use, especially in low frequencies (such as those used in this study). To correct such limitations, our lab is currently conducting a large study using combustible tobacco products, specially designed by the National Institute of Drug Addiction (NIDA) to be identical except for the nicotine content, which will allow us to perform the study in a double-blinded manner.

Lastly, two products elicited subjective and EEG responses in the participants that were not consistent with our hypothesis. General Swedish Snus, with a higher nicotine concentration, produced smaller effects than Hawken Rough, a product with lower nicotine content. We believe that these unexpected results can be explained by differences in the nicotine administration, since Swedish Snus is packed within a sachet, which can delay nicotine absorption rates. Although this pattern of results does not deny our hypothesis, future studies should consider this factor when deciding which products to include.

## Declaration of competing interest

All the authors report that they do not have any conflict of interest in relation with the contents of this manuscript and its implications.

## Figures and Tables

**Table:** 

Subjective factor	Questionnaire	F test paper	P value paper	P-value Adj. Cig use	P-value Adj. years of smokeless products	P-value Adj. frequency of use	P-value Adj. number of products used	P-value Adj. smokeless prod normally used	P-value Adj. all covariates
Total urge to use smokeless tobacco	mQSU-brief	3.77	0.0065	0.0066	0.0066	0.0064	0.0066	0.0065	0.0066
Total withdrawal	mMNWS	4.18	0.0035	0.0034	0.0036	0.0039	0.0035	0.0033	0.0038
Negative affect from withdrawal	mMNWS	3.94	0.0050	0.0049	0.0052	0.0058	0.0050	0.0048	0.0055
Craving from withdrawal	mMNWS	2.53	0.0450	0.0453	0.0466	0.0473	0.0452	0.0452	0.0484
Increased appetite from withdrawal	mMNWS	5.26	0.0007	0.0006	0.0007	0.0007	0.0007	0.0006	0.0007
Total reinforcement	mCES	27.98	<0.0001	<0.0001	<0.0001	<0.0001	<0.0001	<0.0001	<0.0001
Satisfaction	mCES	19.35	<0.0001	<0.0001	<0.0001	<0.0001	<0.0001	<0.0001	<0.0001
Psychological reward	mCES	15.56	<0.0001	<0.0001	<0.0001	<0.0001	<0.0001	<0.0001	<0.0001
Aversion	mCES	13.59	<0.0001	<0.0001	<0.0001	<0.0001	<0.0001	<0.0001	<0.0001
Enjoyment of sensations	mCES	12.81	<0.0001	<0.0001	<0.0001	<0.0001	<0.0001	<0.0001	<0.0001
Reduced craving	mCES	25.49	<0.0001	<0.0001	<0.0001	<0.0001	<0.0001	<0.0001	<0.0001
Total sensory perception	mDSQ	47.69	<0.0001	<0.0001	<0.0001	<0.0001	<0.0001	<0.0001	<0.0001
Liking	mDSQ	15.70	<0.0001	<0.0001	<0.0001	<0.0001	<0.0001	<0.0001	<0.0001
Satisfaction	mDSQ	25.87	<0.0001	<0.0001	<0.0001	<0.0001	<0.0001	<0.0001	<0.0001
Perceived amount of nicotine	mDSQ	40.26	<0.0001	<0.0001	<0.0001	<0.0001	<0.0001	<0.0001	<0.0001
Perceived strength	mDSQ	42.84	<0.0001	<0.0001	<0.0001	<0.0001	<0.0001	<0.0001	<0.0001


Objective Factor	Variable	F Test paper	P Value Paper	P-value Adj. Cig use	P-value Adj. years of smokeless products	P-value Adj. frequency of use	P-value Adj. number of products used	P-value Adj. smokeless prod normally used	P-value Adj. all covariates
Low alpha		16.79	<0.0001	<0.0001	<0.0001	<0.0001	<0.0001	<0.0001	<0.0001
High alpha		5.28	0.0006	0.0006	0.0007	0.0006	0.0006	0.0007	0.0006
Delta		13.69	<0.0001	<0.0001	<0.0001	<0.0001	<0.0001	<0.0001	<0.0001

## Appendix 1

Variables from the Tobacco Use History (TUH) Questionnaire:

• Cigarette use: *TUH7A*. *Do you currently smoke cigarettes?*
• Years of smokeless products used: *TUH2*. *How long have you used smokeless tobacco at this current rate?*
• Type of smokeless product normally used: *TUH4*. *Which brand of smokeless tobacco/chew do you use?*
• Frequency of use: *TUH1*. *How many tins/pouches do you use per week?-Quantity Number of tins or pouches*
• Number of products used per day: Sum of the variables: *TUH5A. Do you currently smoke a pipe?, TUH6A. Do you currently smoke cigars?* and *TUH7A*. *Do you currently smoke cigarettes?*
